# System of Work and Stress-Coping Strategies Used by Nurses of a Polish Hospital during the COVID-19 Pandemic

**DOI:** 10.3390/ijerph20064871

**Published:** 2023-03-10

**Authors:** Beata Haor, Anna Antczak-Komoterska, Justyna Kozyra, Natalia Grączewska, Mariola Głowacka, Monika Biercewicz, Agnieszka Królikowska, Renata Jabłońska, Lech Grzelak

**Affiliations:** 1 Faculty of Health Sciences, State University of Applied Sciences in Wloclawek, 87-800 Wloclawek, Poland; 2 Faculty of Health Sciences, The Mazovian Academy in Plock, 09-402 Plock, Poland; 3 Clinic of Geriatrics, Faculty of Health Science, Collegium Medicum in Bydgoszcz, Nicolaus Copernicus University in Torun, 85-094 Bydgoszcz, Poland; 4 Neurological and Neurosurgical Nursing Department, Faculty of Health Science, Collegium Medicum in Bydgoszcz, Nicolaus Copernicus University in Torun, 85-821 Bydgoszcz, Poland

**Keywords:** stress, nurses, coping, pandemic, COVID-19, burnout

## Abstract

The COVID-19 pandemic contributed to increased stress levels experienced by employees of the healthcare system during their professional activities. The aim of the study was to compare the stress-coping strategies used by nurses in two different systems of work (one shift/two shifts) in a Polish hospital in 2021. The study used the Polish adaptation of the Mini-COPE questionnaire and the authors’ data sheet. The results indicate that regardless of experience or the place and system of work, nurses more often chose problem-focused coping strategies. Conducting screening tests among nurses will help to establish effective strategies for coping with occupational stress, thus preventing professional burnout.

## 1. Introduction

Working during a pandemic is especially challenging for nurses. In this scenario, professional activities are undertaken that go beyond standard routine or applied patterns of conduct, which are also practised by smaller nursing teams under the pressure of time and the risk of loss of life or failing health [1]. The above observations were confirmed by earlier research results from the period of the SARS epidemic [2,3].

The epidemic caused by SARS-CoV-2 viral infections was announced in Poland on 20 March 2020. Measures against the epidemic were introduced, and a state of emergency was declared on 16 May 2022. The successive waves of the pandemic and the announced lockdown periods had a significant impact on the lives of Polish citizens, as well as residents of other European countries. This was due to the cyclical introduction of a rigorous sanitary regime and restrictions on private and professional contacts in education, access to trade, services, national and international communication, etc., in line with the increasing number of cases and deaths caused by SARS-CoV-2 infections. Medical staff, including nurses, who provided services under the completely new and unprecedented conditions of this sanitary regime, found themselves in a unique situation, exposed to the possibility of coronavirus infection and potentially death. During successive waves of the pandemic, nurses worked in a dynamically changing regime in temporary hospitals or hospitals that were closed to visitors. They also remained in periods of isolation from their families or other members of the therapeutic team. They took care of patients in critical conditions and those who were dying, who in the last moments of their lives were isolated from their families and loved ones. The professional activities of nurses were accompanied by completely new diagnostic and therapeutic procedures and a period of uncertainty while waiting for the first doses of effective vaccines. In the autumn of 2021, when this study was conducted, nurses carried out professional activities during the fourth wave of the coronavirus pandemic. This occurred after a period of easing some sanitary requirements and other restrictions, as well as introducing vaccinations into the population [4].

The period of the COVID-19 pandemic has the features of a crisis, which, according to Clark, is determined by such elements as time pressure, potential threat and surprise, and danger due to the circumstances in which it occurs [1,5]. In turn, the consequence of critical events are changes in the adaptation mechanisms in the human–environment–group–environment system [6]. Therefore, people facing a crisis experience acute or chronic stress, emotional tension, and a sense of uncertainty due to the loss of control over routine activities, which, in turn, forces them to make changes to their current behaviour, i.e., adopting coping strategies [1,7]. Eustress has a motivating effect and stimulates an individual to develop and change. Distress, on the other hand, has a negative or even destructive effect and is difficult to control [8]. Research conducted during the COVID-19 pandemic confirms that Polish nurses experienced severe stress and perceived the pandemic as an important threat to their health and safety [9,10]. This means that during the COVID-19 pandemic, nurses, along with other healthcare workers, experienced distress, which was mainly related to the experience of fear, risk related to treating an infected patient, numerous deaths of patients and staff, uncontrolled emergency situations, and long work shifts [10].

Coping is treated as a process and style of strategy that is specific to an individual [11]. It is assumed that these beliefs and behaviours are a response to a particular situation [12]. A number of studies on coping strategies can be found in the literature [1,13,14]. Endler and Parker indicate three styles of coping with stress: problem solving, emotions, and avoidance [15]. Problem-solving strategies include planning, instrumental counselling, seeking support or information, or confrontation. Strategies focused on emotional regulation include avoidance, withdrawal, affective venting, talking about emotions, and persistently thinking about a problem. Paradoxically, however, these strategies are conducive to positive re-evaluation and the search for emotional social support. The avoidance of strategies includes a loss of control or escape reactions [1]. In coping with the distress accompanying professional work as a nurse during a pandemic, it seems constructive to use strategies focused on the task at hand.

The COVID-19 pandemic had a significant impact on the mental health of nurses due to high levels of stress caused [16,17]. The pandemic certainly exacerbated the currently growing problem of professional burnout, which is defined as a psychological syndrome of emotional exhaustion, depersonalisation, and a reduced sense of personal achievement. It is specific to people who work in teams as part of their professional activities [18,19]. Meta-analyses of research findings confirm that the COVID-19 pandemic has contributed to an increase in burnout rates in groups of medical workers, especially nurses [20]. Polish nurses, regardless of the specialties presented, showed signs of occupational burnout even before the pandemic period [21,22]. This is also confirmed by the results of the RN4CAST study conducted in 12 European countries, including Poland. These results indicated high rates of job dissatisfaction and nurses’ intention to leave their current positions [23].

During the pandemic, nurses experienced not only severe stress but also increased levels of anxiety, depression, insomnia, and chronic fatigue [24,25,26]. This, combined with insufficient skills to cope with stress, the complexity of procedures carried out in the workplace, and a lack of support from colleagues, may lead to the emergence or intensification of symptoms of burnout syndrome [22]. 

The aim of the study was to compare the stress-coping strategies used by nurses in two different systems of work (one shift/two shifts) in a Polish hospital in 2021. Conducting screening tests in this area will help to support nursing teams in the situation of a public health crisis and prevent burnout syndrome [17,24]. Reliable information support, reductions in stress and tension, contact with family, and access to personal protective equipment are all essential for the welfare of nurses. Due to many hours of continuous work, they should have guaranteed time to rest and achieve their daily goals [24].

## 2. Materials and Methods

### 2.1. Participants

The study was conducted from November to December 2021 on 447 nurses employed at the Provincial Specialist Hospital named after the blessed priest Jerzy Popieluszko in Wloclawek (Poland). They were informed about the purpose of the study, its anonymous nature, and voluntary participation. After obtaining confirmation of consent to participate in the study, the respondents were informed that they could download questionnaires at any time from designated places at work. After completing them at any time, subjects could drop the questionnaires into a sealed urn that was set up in the department. Only fully completed questionnaires qualified for statistical analysis, of which there were 100. The inclusion criteria for this study were as follows: consent to participate in an anonymous study and employment in a hospital in Wloclawek as a nurse. On the other hand, the exclusion criteria were a lack of consent to an anonymous study and employment in the aforementioned hospital in a position other than as a nurse.

### 2.2. Measures

The study used the Mini-COPE Inventory, which is a shortened version of the Coping Orientations to Problems Experienced (COPE) developed by Carver [27]. The Polish adaptation of the tool was created by Juczyński and Ogińska-Bulik [28]. The Mini-COPE was preceded by an original development of a metric containing a total of ten questions regarding respondents’ socio-demographic data (age, gender, place of residence, marital status, and education), their professional situation (experience in the profession, type of branch in which they are employed, and system of work), and the respondents’ opinions on whether the period of the COVID-19 pandemic contributed to increased stress at work, as well as whether they dealt with it better during the pandemic than beforehand. The Polish version of Mini-COPE consists of 28 statements that refer to 14 strategies for coping with stress. Each strategy contains two such statements. The respondent selects one of the four answers in each statement, which have the following scores: 0—I almost never do this; 1—I rarely do this; 2—I often do this; and 3—I almost always do this. Each strategy was independently assessed by adding the points together and dividing the result by 2. In each strategy, the score ranges from 1 to 3. The higher the score, the more often the respondent uses a given strategy. The strategies are divided into four categories and their corresponding factors, such as active coping (active coping, planning, and positive re-evaluation); helplessness (using psychoactive substances, cessation of activities, and self-blame); seeking support (seeking emotional support and seeking instrumental support); and avoidance behaviours (doing something else, denying, and venting). In turn, the following strategies of turning to religion, acceptance, and sense of humour are separate elements [28]. The duration of the test is 10 min. The Polish version of Mini-COPE is available free of charge on the website of the Laboratory of Psychological Tests (Poland) [29].

### 2.3. Procedure and Ethical Considerations

The study was conducted in accordance with the Declaration of Helsinki and approved by the Bioethics Committee at the National Academy of Applied Sciences in Wloclawek (Resolution No. 40/21 of 22 November 2021). Consent to conduct the study was also granted by the director of the Provincial Specialist Hospital, named after the blessed priest Jerzy Popieluszko in Wloclawek, where the nurses participating in the study were employed (consent was obtained on 7 July 2021).

### 2.4. Statistical Analysis

The research was subjected to descriptive, graphical, and statistical analysis. All calculations were performed using the SPSS Statistics 21.0 statistical package. The ANOVA test and Student’s *t*-test were used to examine the statistical relationship between the analysed features. Descriptive statistics for continuous variables were expressed as mean (M), standard deviation (SD), results of the analysis of variance (F), and results of Student’s *t*-test (t). Categorical variables were expressed as frequency (*n*). A 5% risk of inference error was assumed. The probability value (*p*) was considered statistically significant when *p* < 0.05.

## 3. Results

There were 100 nurses in the analysed group: 87 women (87%) and 13 men (13%). The largest group were respondents aged 40–59 (*n* = 53, 53%) and aged 21–39 (*n* = 38, 38%). The smallest group comprised participants aged 60 and over (*n* = 9; 9%). Sixty-six respondents (66%) lived in cities, and thirty-four (34%) lived in the countryside. Forty-two respondents (42%) had completed bachelor’s studies in nursing, and thirteen (13%) had completed master’s studies. On the other hand, 27 medical school graduates (27%) and 18 medical high school graduates (18%) had obtained a nursing diploma under the vocational education system in force in Poland before integration into the European Union. In terms of marital status, 44 people (44%) were married, 23 declared being single (23%), 21 were divorced (21%), and 12 respondents (12%) were widows/widowers. The largest group were respondents with between 13 months and 10 years of work experience (*n* = 13; 33%). In turn, 32 respondents (32%) had worked as nurses for 11–25 years. Respondents with work experience of over 26 years constituted another group of respondents (*n* = 30; 30%). The fewest respondents (*n* = 5; 5%) had up to 12 months of work experience. Most nurses were employed in medical wards (*n* = 35; 35%): 28 respondents (28%) worked in surgical wards, 15 in the hospital emergency department (15%), and 14 in specialist clinics (14%). In turn, eight respondents (*n* = 8) were employed in other locations, such as a dialysis station, an invasive cardiology laboratory, and an imaging examination laboratory. In terms of work systems, 67 respondents (67%) worked in a two-shift system and 33 in a single-shift system (33%).

The strategies of coping with stress used by nurses are presented in Table 1.

The strategies of coping with stress most often used by the respondents were acceptance, expressed by accepting the situation and learning how to live with it (M = 4.16; SD = 1.16) and active coping by taking actions to improve the situation (M = 4.06; SD = 1.15). High average scores also concerned the selection of such strategies as occupying yourself with something else by performing various activities so as not to think about stressful events (M = 3.98; SD = 1.36) and planning, i.e., thinking about what should be undertaken to solve the problem (M = 3.85; SD = 1.21). The lowest average scores were obtained by the respondents using stress-coping strategies such as a sense of humour based on joking and treating the situation as fun (M = 2.44; SD = 1.53) and taking psychoactive substances to alleviate unpleasant emotions (M = 1.56; SD = 1.69).

The selected strategies of coping with stress by nurses significantly differ depending on their work system, which is presented in Table 2. 

The statistical analysis showed significant differences between the respondents’ work systems and their selection of such stress-coping strategies such as active coping, turning to religion, and seeking instrumental support. For each of the mentioned strategies, the respondents working in a single-shift system obtained higher results than those employed in a two-shift system. The respondents from the single-shift work system compared to the respondents from the two-shift work system used strategies such as active coping (average number of points = 4.39 > average number of points = 3.90), turning to religion (average number of points = 3.88 > average number of points = 2.99), and seeking instrumental support (average number of points = 4.45 > average number of points = 3.54) significantly more often. No significant differences were found in other stress-coping strategies depending on the nurses’ work systems. Nevertheless, the respondents working in one shift most often undertook actions within the strategies of seeking instrumental support (average number of points = 4.45) and active coping (average number of points = 4.39). However, they rarely used the cessation strategy (average number of points = 2.27) and psychoactive substances (average number of points = 1.09). In turn, respondents employed in a two-shift system to cope with stress most often used the following strategies: acceptance (average number of points = 4.06) and doing something else (average number of points = 3.94). The least frequently used strategies were cessation of activities (average number of points = 2.69) and using psychoactive substances (average number of points = 1.79). 

The selected strategies of coping with stress by nurses significantly differ depending on their experience, which is presented in Table 3.

Statistical analysis showed significant differences between the experience of the respondents and their choice of strategy of coping with stress such as acceptance. Respondents with work experience of 26 years and more used the above strategy significantly more often (average number of points = 4.73) compared to the rest of the respondents employed in the profession for 11–25 years (average number of points = 3.84), 13 months to 10 years (average number of points = 3.91), and with up to 12 months of work experience (average number of points = 4.40). In terms of other strategies, there were no significant differences in the use of strategies depending on levels of experience. Nevertheless, nursing staff with work experience of up to 12 months in a stressful situation most often undertook actions using the following strategies: acceptance, planning, and active coping (average number of points 4.40 each). The least frequently used were the cessation of activities (average number of points = 1.00) and taking psychoactive substances (average number of points = 0.80). Respondents who worked between 13 months and 10 years most often used active coping strategies (average number of points = 3.94), acceptance, and seeking instrumental support (average number of points 3.91 each). The least frequently used strategies were a sense of humour (average number of points = 2.06) and taking psychoactive substances (average number of points = 1.18). People with 11 to 25 years of work experience most often used the following strategies: planning (average number of points = 4.16) and seeking emotional support (average number of points = 4.09). They used the sense of humour strategy to the smallest extent (average number of points = 2.41) and the use of psychoactive substances (average number of points = 1.78). People working in the profession for more than 26 years reported that they most often used the strategies of acceptance (average number of points = 4.73) and taking care of something else (average number of points = 4.40). The least frequently used strategies were cessation of activities (average number of points = 2.73) and taking psychoactive substances (average number of points = 1.87). 

A statistical analysis showed no significant relationship between the respondents’ workplace and stress-coping strategies. People working in conservative wards reported that they most often used the strategies of acceptance (average number of points = 4.17) and planning (average number of points = 4.03). They rarely used strategies based on a sense of humour (average number of points = 2.29) and the use of psychoactive substances (average number of points = 1.86). Those employed in surgical departments most often relied on the following strategies: active coping (average number of points = 4.43) and acceptance (average number of points = 4.11). In turn, they least often used the following strategies: a sense of humour, cessation of activities (average number of points 2.11 each), and taking psychoactive substances (average number of points = 1.32). Employees of the hospital emergency department reported that their behaviour in stressful situations was most often based on the following strategies: doing something else (average number of points = 4.13) and acceptance (average number of points = 3.93). They rarely used the strategies of denial (average number of points = 2.73) and psychoactive substances (average number of points = 2.07). Nursing staff working in specialist clinics most often used the following strategies: seeking instrumental support (average number of points = 4.57) and emotional support and an acceptance strategy (average number of points = 4.50 each). The sense of humour strategy (average number of points = 2.43) and psychoactive substances (average number of points = 1.36) were used less frequently. People employed in other places within the hospital reported that they most often used the following strategies in stressful situations: acceptance (average number of points = 4.13) and positive re-evaluation (average number of points = 4.00). In turn, the least frequently used strategies were cessation of activities (average number of points = 1.63) and taking psychoactive substances (average number of points = 0.50).

The largest group (*n* = 70; 70%) were the respondents who confirmed that the timing of the COVID-19 pandemic increased stress levels at work. Fifteen respondents (15%) partially shared the above opinion, and nine did not confirm this (9%). In turn, six respondents (6%) indicated that they had no opinion on this subject. However, the statistical analysis did not show any significant differences between the opinion of the respondents on whether the COVID-19 pandemic contributed to their increased perception and experience of stress at work and stress-coping strategies. Nevertheless, the respondents who claimed that the pandemic caused them an increased sense of stress, most often undertook coping strategies such as acceptance (average number of points = 4.14) and active coping (average number of points = 4.11). The strategies of a sense of humour (average of l point = 2.40) and taking psychoactive substances (average number of points = 1.51) were used the least often. Respondents whose stress levels did not increase due to the pandemic used the following strategies: acceptance (average number of points = 4.78) and positive re-evaluation (average number of points = 4.56). They rarely used strategies of denial (average number of points = 2.56) and taking psychoactive substances (average number of points = 2.33). People for whom the pandemic contributed to a partial increase in stress levels often used the following strategies: acceptance (average number of points = 4.07) and doing something else (average number of points = 4.00). On the other hand, the strategies of a sense of humour (average number of points = 2.47) and the use of psychoactive drugs (average number of points = 1.33) were used less willingly. Respondents who had no opinion on the impact of the pandemic on their stress reported that they usually adopt the strategies of seeking emotional support (average number of points = 4.67) and acceptance (average number of points = 4.50). In turn, they least often use strategies such as the cessation of activities (average number of points = 1.88) and taking psychoactive substances (average number of points = 1.50).

The choice of strategy was significantly differentiated depending on the respondents’ opinions on whether they coped with stress at work better during the COVID-19 pandemic than in the pre-pandemic period, as presented in Table 4.

Statistical analysis showed a significant relationship (*p* = 0.000) between the respondents’ opinions on whether they coped with stress at work better during the COVID-19 pandemic compared to the pre-pandemic period and the following strategies: active coping, acceptance, turning to religion, seeking instrumental support, and doing something else. Respondents who confirmed that they coped better with stress during the pandemic (*n* = 33) used the following methods more often than others: active coping (average number of points = 4.52), acceptance (average number of points = 4.52), seeking instrumental support (average number of points = 4.42), and doing something else (average number of points = 4.36). The respondents who believed that they partly coped better with stress at work during the COVID-19 pandemic also often used the strategies of acceptance (average number of points = 4.25) and seeking instrumental support (average number of points = 3.96). The respondents who claimed that the pandemic period had no impact on their ability to cope with stress in the workplace (*n* = 30) used the strategy of turning to religion more often than other respondents (average number of points = 3.77). The respondents who had no opinion on this subject often used the strategy of active coping (average number of points = 4.15) and doing something else (average number of points = 3.85). There were no statistically significant differences in the remaining strategies. Nevertheless, nurses coped better with stress during the pandemic than beforehand under the following strategies: acceptance (average number of points = 4.52) and active coping (average number of points = 4.52). In turn, they rarely used strategies based on a sense of humour (average number of points = 2.67) and taking psychoactive substances (average number of points = 1.76). Respondents denying the statement that they coped better with stress during the pandemic than before its occurrence more often based their actions on the following strategies: acceptance (average number of points = 4.87) and turning to religion (average number of points = 4.77). On the other hand, they rarely used the following strategies: a sense of humour (average number of points = 2.43) and taking psychoactive substances (average number of points = 1.63). Respondents who indicated that they partly coped with stress at work better during the pandemic reported that they were more willing to base their behaviour on the following strategies: acceptance (average number of points = 4.45) and planning (average number of points = 4.04). As with other respondents, they rarely used the strategy of denial (average number of points = 2.00) and taking psychoactive substances (average number of points = 1.17). Respondents who had no opinion on whether they coped better with stress during the pandemic than before its occurrence most often used the following strategies: seeking instrumental support (average number of points = 4.23) and active coping (average number of points = 4.15). In turn, they rarely used the following strategies: a sense of humour (average number of points = 2.23) and taking psychoactive substances (average number of points = 1.62).

## 4. Discussion

### 4.1. Coping with Stress

The results of this study indicate that the stress-coping strategies most often used by nurses at work were acceptance and active coping, and a sense of humour and taking psychoactive substances were the least used strategies.

Compared to those employed in a two-shift system, people employed in a single-shift system chose the following strategies significantly more often: active coping, turning to religion, and seeking instrumental support. No significant differences were found in other strategies of coping with stress depending on the nurses’ work system. Nevertheless, the most frequently indicated strategies included acceptance, active coping, seeking instrumental support, and doing something else. Regardless of the work system, the respondents most often used psychoactive substances and ceased activities. 

No significant relationship was found between the workplace of the respondents and the strategies of coping with stress. However, regardless of the place of work, the respondents most often used the following strategies: acceptance, planning, active coping, dealing with something else, instrumental and emotional support, and positive re-evaluation. They used the following strategies the least: a sense of humour, taking psychoactive substances, cessation of actions, and denial. 

A significant relationship between the experience of the respondents and the strategy of coping with stress through acceptance was also confirmed. Respondents with the longest work experience (of 26 years or more) used the above strategy significantly more often in stressful situations compared to other respondents less experience. However, regardless of experience, the respondents more often used active coping, acceptance, instrumental support, planning, emotional support, or doing something else. They used the strategies of a sense of humour and the use of psychoactive substances least often.

The meta-analysis by Kwak et al. on research conducted before the COVID-19 pandemic among Polish nurses, which concerned strategies for coping with stress, is subject to differentiation due to the research methods used. Most often, however, the results indicate that these nurses tend to cope with stress through a style focused on the task. The authors who used the COPE questionnaire in their research confirm that nurses most often used the strategy of active coping and seeking support in a stressful situation [30]. Additionally, other studies conducted in the period preceding the pandemic confirmed that, among the strategies of coping with stress, Polish nurses more often chose active coping, planning, dealing with other activities, seeking emotional support, and positive re-evaluation. Among the less popular strategies were denial, sense of humour, cessation of activities, taking psychoactive substances, and seeking specialist help [1,31,32,33]. 

This study, conducted during the COVID-19 pandemic, confirms the more frequent use of strategies based on tasks and emotions as opposed to avoiding difficult situations. This is indicative of the strategies that experienced Polish nurses could use when struggling with crisis situations during the pandemic. Coping with stress can be phasic. The current study looked at nursing teams that had been struggling with the COVID-19 pandemic for a year. It is based on both the experience from before the pandemic period and the actions taken during a year of the pandemic.

In this study, the largest group of respondents were those who confirmed that the timing of the COVID-19 pandemic caused increased stress levels at work. Statistical analysis, however, did not reveal any significant differences between the opinion of the respondents on whether the period of the COVID-19 pandemic contributed to their increased perception and experience of stress at work and the choice of stress-coping strategies. Nevertheless, the respondents who confirmed or partially agreed that the pandemic caused increased feeling of stress most often undertook coping strategies such as acceptance, active coping, and doing something else. The strategies of a sense of humour and taking psychoactive substances were used the least often. In turn, respondents whose stress levels did not increase in connection with the pandemic or those who were unable to clearly define their position in this regard most often used the following strategies: acceptance, positive re-evaluation, and emotional support. They rarely used the strategies of denial, substance use, and disengagement. 

Statistical analysis showed a significant relationship between respondents’ opinions on whether they coped with stress at work better during the COVID-19 pandemic than in the pre-pandemic period using the following strategies: active coping, acceptance, turning to religion, seeking instrumental support, and doing something else. Respondents who confirmed or partially agreed that they coped better with stress during the pandemic most often used the following strategies: active coping, acceptance, seeking instrumental support, and doing something else. In turn, the respondents who claimed or were unsure that the pandemic period had no impact on coping with stress in the workplace used the following strategies more often than other respondents: turning to religion, active coping, and doing something else. Regardless of the opinions on better coping with stress during the pandemic or before its occurrence, the respondents least often indicated the following strategies: a sense of humour and the use of psychoactive substances.

Research conducted among Polish nurses during the COVID-19 pandemic confirms that the vast majority of them experienced increased symptoms of stress [10,34], largely due to changes in the existing workplace organisation and the fear of transmitting the disease to their family. However, after a year of working during the pandemic, stress remained at medium and low levels, which may indicate a phase of adaptation to the public health crisis [34]. 

Studies of Jordanian nurses during the COVID-19 pandemic also confirm a particular exposure to acute stress. This may contribute to the development of PTSD (post-traumatic stress disorder). Self-efficacy, older age, and thus longer work experience may be important factors that prevent high levels of stress [17].

Studies of nurses during the COVID-19 pandemic in China indicate that, compared to nursing students and due to their professional experience, they experienced more intense reactions to the COVID-19 pandemic. However, they used more strategies focused on the problem than on emotions [35,36]. Coping in a crisis situation may, therefore, be characterised by phasing, which is also confirmed by the results of research conducted among nursing management staff in Taiwan during the SARS epidemic. The use of emotion-based strategies in the initial phase of the epidemic allowed problem strategies to be used later [37]. 

Ślusarz et al., in a meta-analysis of studies conducted in different countries during the COVID-19 pandemic, confirmed the co-occurrence of many predictors of occupational burnout among nurses of various specialties. These included exhaustion, depressive disorders, fear, anxiety, etc. [20].

### 4.2. Limitations

Our study has some limitations. Firstly, the presented results come from a study that is based on the subjective assessment of nurses’ feelings. However, the study used a standardised research tool. Secondly, the group participating in the study was characterised by a small number (100 people out of 447 nurses employed in the hospital). This was due to the simple random selection for the study, the exclusion of incomplete questionnaires from further analyses, and the voluntary participation of people in the study. Another limitation is that the study included respondents from one hospital. In addition, in the presentation of the results, we referred to the opinions of respondents expressed in 2021, i.e., the next year of the pandemic. Therefore, it is difficult to make the fully obtained results generalisable to all nurses working in this hospital and the general population of the whole country. However, our results are consistent with those of other studies conducted on nurses from Poland and other countries. Despite these limitations, the results of our study may be a starting point for further research on the use of stress-coping mechanisms by nurses during the COVID-19 pandemic, especially research on strategies to prevent burnout, which may intensify during the epidemic.

## 5. Conclusions

Nurses are one of the occupational groups that are the most exposed to stress. Among numerous stress factors affecting nurses, the most prevalent are direct contact with patients and family, cooperation with members of the therapeutic team, and work in an environment that has negative connotations for human life. The COVID-19 pandemic, as a situation with features of a public health crisis, significantly contributed to the intensification of the negative consequences of acute stress experienced by nurses. The scope of professional responsibility related to the performed tasks, the pressure of time, reliability, and meeting social expectations during the difficult period of the pandemic significantly influenced the coping strategies of nurses during the pandemic. The consequences of experiencing acute stress are particularly noticeable in the mental health of nurses. They may even promote the development of PTSD. Conducting screening tests among nurses will allow for the design of optimal psychological support, the preparation of training programs in order to build effective solutions in the field of strategies for coping with occupational stress, and the prevention of occupational burnout.

## Figures and Tables

**Table 1:** Strategies of coping with stress among respondents.

Strategy Name		
	M	SD
Active coping	4.06	1.15
Planning	3.85	1.21
Positive re-evaluation	3.73	1.26
Acceptance	4.16	1.16
Sense of humour	2.44	1.53
Turning to religion	3.28	1.63
Seeking emotional support	3.82	1.34
Seeking instrumental support	3.84	1.41
Doing something else	3.98	1.36
Denial	2.87	1.59
Venting	3.42	1.46
Using psychoactive substances	1.56	1.69
Cessation of activities	2.55	1.55
Self-blame	3.02	1.47

**Table 2:** Strategies of coping with stress used by nurses depending on the system of their work.

Work System:	One Shift			Two Shifts (Day/Night)				
	Mean	*n*	SD	Mean	*n*	SD	t	*p*
Strategies								
Active coping	4.39	33	1.06	3.90	67	1.17	4.27	0.041
Planning	3.79	33	1.52	3.88	67	1.04	0.13	0.720
Positive re-evaluation	3.48	33	1.28	3.85	67	1.25	1.88	0.174
Acceptance	4.36	33	1.14	4.06	67	1.17	1.52	0.220
Sense of humour	2.36	33	1.41	2.48	67	1.60	0.12	0.729
Turning to religion	3.88	33	1.71	2.99	67	1.51	7.08	0.009
Seeking emotional support	4.18	33	1.40	3.64	67	1.29	3.67	0.058
Seeking instrumental support	4.45	33	1.33	3.54	67	1.36	10.19	0.002
Doing something else	4.06	33	1.32	3.94	67	1.38	0.17	0.679
Denial	2.64	33	1.65	2.99	67	1.56	1.06	0.306
Venting	3.33	33	1.49	3.46	67	1.46	0.17	0.680
Using psychoactive substances	1.09	33	1.38	1.79	67	1.79	3.90	0.051
Cessation of activities	2.27	33	1.68	2.69	67	1.48	1.58	0.212
Self-blame	2.67	33	1.49	3.19	67	1.44	2.90	0.092

**Table 3:** Strategies of coping with stress used by nurses depending on their experience.

Work Experience:	11–25 Years			13 Months to 10 Years			26 Years or More			Up to 12 Months				
	Mean	*n*	SD	Mean	*n*	SD	Mean	*n*	SD	Mean	*n*	SD	F	*p*
Strategies														
Active coping	4.03	32	1.31	3.94	33	1.12	4.17	30	1.09	4.40	5	0.89	0.35	0.789
Planning	4.16	32	1.05	3.70	33	1.26	3.60	30	1.28	4.40	5	1.14	1.67	0.180
Positive re-evaluation	3.59	32	1.24	3.58	33	1.28	3.97	30	1.19	4.20	5	1.79	0.87	0.461
Acceptance	3.84	32	1.25	3.91	33	1.04	4.73	30	1.05	4.40	5	0.89	4.18	0.008
Sense of humour	2.41	32	1.58	2.06	33	1.41	2.90	30	1.47	2.40	5	2.07	1.61	0.192
Turning to religion	3.38	32	1.43	3.24	33	1.66	3.33	30	1.67	2.60	5	2.61	0.34	0.799
Seeking emotional support	4.09	32	1.17	3.79	33	1.43	3.57	30	1.36	3.80	5	1.79	0.80	0.497
Seeking instrumental support	4.06	32	1.56	3.91	33	1.31	3.57	30	1.19	3.60	5	2.30	0.71	0.550
Doing something else	4.06	32	1.52	3.67	33	1.05	4.40	30	1.40	3.00	5	1.00	2.58	0.058
Denial	2.88	32	1.43	2.76	33	1.79	3.27	30	1.44	1.20	5	1.30	2.63	0.055
Venting	3.41	32	1.36	3.15	33	1.48	3.83	30	1.60	2.80	5	0.45	1.49	0.223
Using psychoactive substances	1.78	32	1.74	1.18	33	1.53	1.87	30	1.81	0.80	5	1.30	1.42	0.242
Cessation of activities	2.81	32	1.49	2.36	33	1.37	2.73	30	1.68	1.00	5	1.73	2.36	0.077
Self-blame	3.03	32	1.12	3.03	33	1.65	3.17	30	1.58	2.00	5	1.58	0.90	0.444

**Table 4:** The influence of the COVID-19 pandemic on coping with stress by the respondents and strategies of coping with stress.

In Your Opinion, during the COVID-19 Pandemic, Did You Cope with Stress at Work Better than before the Pandemic?														
	Yes			No			Partly			No Opinion				
	Mean	*n*	SD	Mean	*n*	SD	Mean	*n*	SD	Mean	*n*	SD	F	*p*
Strategies														
Active coping	4.52	33	1.23	3.70	30	1.26	3.83	24	0.82	4.15	13	0.90	3.23	0.026
Planning	4.06	33	1.37	3.57	30	1.41	4.04	24	0.86	3.62	13	0.65	1.26	0.294
Positive re-evaluation	4.09	33	1.40	3.57	30	1.22	3.71	24	1.16	3.23	13	1.01	1.79	0.154
Acceptance	4.52	33	1.28	3.87	30	1.11	4.25	24	1.03	3.77	13	1.01	2.29	0.083
Sense of humour	2.67	33	1.53	2.43	30	1.30	2.25	24	1.73	2.23	13	1.74	0.44	0.727
Turning to religion	3.55	33	1.44	3.77	30	1.74	2.63	24	1.64	2.69	13	1.38	3.26	0.025
Seeking emotional support	4.03	33	1.31	3.73	30	1.34	3.42	24	1.44	4.23	13	1.17	1.46	0.231
Seeking instrumental support	4.42	33	1.32	3.13	30	1.38	3.96	24	1.43	3.77	13	1.01	4.99	0.003
Doing something else	4.36	33	1.34	4.03	30	1.27	3.46	24	1.28	3.85	13	1.52	2.20	0.093
Denial	2.82	33	1.63	3.17	30	1.37	2.83	24	1.79	2.38	13	1.66	0.76	0.520
Venting	3.58	33	1.41	3.40	30	1.48	3.29	24	1.71	3.31	13	1.18	0.21	0.891
Using psychoactive substances	1.76	33	1.68	1.63	30	1.61	1.17	24	1.52	1.62	13	2.22	0.60	0.617
Cessation of activities	2.82	33	1.72	2.73	30	1.39	2.00	24	1.50	2.46	13	1.45	1.51	0.217
Self-blame	3.15	33	1.39	3.03	30	1.65	2.92	24	1.64	2.85	13	0.90	0.18	0.907

## Data Availability

Data are available upon reasonable request.
